# The relationship between the 1200 m shuttle test and 40 m sprint test performance and distances covered in English Premier League matches: A retrospective two season study

**DOI:** 10.5114/biolsport.2025.142641

**Published:** 2024-10-02

**Authors:** Ronan Kavanagh, Matteo Matteotti, Rafael Oliveira, Kevin McDaid, Jill Alexander, Damian Harper, Piotr Zmijewski, David Rhodes, Ryland Morgans

**Affiliations:** 1 Performance and Analytics Department, Parma Calcio 1913, 43121 Parma, Italy; 2 Football Performance Hub, Institute of Coaching and Performance, University of Central Lancashire, Preston, UK; 3 Research Center in Sport Sciences, Health Sciences and Human Development (CIDESD), Santarém Polytechnic University, Av. Dr. Mário Soares, 2040-413 Rio Maior, Portugal; 4 Santarém Polytechnic University, School of Sport, Av. Dr. Mário Soares, 2040-413 Rio Maior, Portugal; 5 Applied Data Analytics Research Group, Dundalk Institute of Technology, Louth, Ireland; 6 Jozef Pilsudski University of Physical Education in Warsaw, 00-809 Warsaw, Poland; 7 Research and Development Center Legia Lab, Legia Warszawa, Poland; 8 Human Performance Department, Burnley FC, Burnley, UK; 9 School of Sport and Health Sciences, Cardiff Metropolitan University, Cardiff, UK

**Keywords:** Maximal aerobic speed, Anaerobic speed reserve, Training, Soccer, English Premier League players

## Abstract

To identify a relationship between the 1200 m shuttle test and 40 m sprint test performance with distances covered at varying intensities in English Premier League (EPL) matches. A squad (n = 21) of full-time professional 1^st^ team male football players (age 29.8 ± 3.4 years; height 183.7 ± 5.2 cm; weight 83.7 ± 6.9 kg) participated in this study. League match data from the 2019–20 and 2020–21 seasons were recorded and analysed via an Optical Tracking System (OTS) (Second Spectrum^®^, Los Angeles, USA) to report physical match performance data. Average velocity during the 1200 m shuttle test (V1.2ST) was calculated, while Peak sprinting speed (PSS) was estimated using a 40 m maximal sprint. ASR1.2ST was established by subtracting V1.2ST from PSS. The relationship between V1.2ST, 30%ASR1.2ST and distances covered at varying intensities in EPL matches was assessed by a series of independent Linear Mixed Effects (LME) models. Although not statistically significant, for every unit increase in V1.2ST, there was an increase of 1032 m in distance covered, (p = 0.07). A single unit increase in 30%ASR1.2ST is associated with a significant increase of 495 m in high-speed running distance (> 5.5 m · s^−1^) (p = 0.02). While for each unit increase in 30%ASR1.2ST, sprint distance (> 7 m · s^−1^) covered significantly increased by 209 m (p = 0.02). In conclusion, high levels of physical fitness such as V1.2ST and 30%ASR1.2ST derived from the 1200 m shuttle and 40 m sprint tests can improve match running performance in elite soccer. Knowledge of this information allows practitioners to tailor training load based on each players individual characteristics, potentially increasing performance.

## INTRODUCTION

The physiological demands of soccer are intermittent by nature [1]. Players alternate between short high-intensity multidirectional efforts and longer periods of low-intensity activity [2, 3]. Quantification of running volumes and intensities performed during match-play are essential for the prescription of appropriate training to optimally prepare players for game demands [4]. When match-play physical demands are examined, load is often characterised by graduated speed thresholds ranging from motionless standing to maximal sprinting [5]. Recent literature has paid particular attention to these high velocity physical metrics to guide training, optimise performance and reduce injury risk [6, 7]. Higher levels of aerobic fitness have been shown to improve recovery time and the ability to perform more high-intensity soccer activities [8]. Aerobic fitness has previously been utilised to differentiate between various compeititve levels [9], while successful teams perform more high-intensity match-play activities than unsuccessful teams when in possession of the ball [8]. This highlights the practical importance of implementing a valid measure that characterises the functional limits of aerobic endurance and sprint capacity [10]. Such information may allow a more precise prescription of running-based exercises required for the individual player [11].

Maximal Aerobic Speed (MAS) is the lowest speed at which VO_2_max may occur [12]. One of the benefits of MAS as a measure of aerobic fitness is the ease at which practitioners can assess large groups of athletes without any expensive equipment required [13]. Time spent above MAS has been shown to correlate (r = 0.77) with improvements in aerobic fitness [14]. Moreover, the distance covered above MAS can differentiate between various performance levels in professional soccer [13]. Swaby et al. [15] observed a strong relationship (r = 0.75) between MAS and distance covered during match-play in professional rugby union players, similar to the findings reported in elite level soccer players [16]. Indeed, knowledge of the relationship between MAS and total distance covered during English Premier League (EPL) matches may inform post-match recovery protocols and team selection for future games. Numerous field-based tests have been developed that significantly correlate with field and laboratory-based MAS tests [17, 18]. The 1200 m shuttle test has been described as reasonable alternative method to measure change in intermittent sports endurance performance [19].

The difference between MAS and Maximal Sprinting Speed (MSS) has been previously quantified as the Anaerobic Speed Reserve (ASR) [20]. The analysis of distance covered above ASR has recently been identified as a reliable method to provide appropriate contextual training prescription [21]. Anaerobic Speed Reserve represents a time-efficient practical field-based construct [22] and has been used to better understand mechanical limits of 800 m runners in addition to tracking progress in training [23]. Ortiz et al. [24] demonstrated that soccer players with higher ASR have a greater absolute sprint performance. In the absence of valid and reliable measures of anaerobic metabolism, ASR provides an estimated value for practitioners to increase an understanding of athlete locomotor profile development [25]. The use of 30% ASR as opposed to ASR alone combines MAS and ASR to ensure that a reduction in MAS would not result in an increase in the ASR measure used [10].

Previous evidence suggests that straight-line sprinting is the most frequent powerful action leading to goals and assists in professional football [26]. The ability to identify the capacity to perform highintensity actions repeatedly during matches may provide a competitive advantage for practitioners and coaches to apply to player and team selection, style and system of play and tactical strategies when in and out of possession and during transitional moments [27]. Therefore, the aim of this study is to identify a relationship between the 1200 m shuttle test and 40 m sprint test performance with distances covered at varying intensities in EPL matches. The study hypothesis is that higher test values derived from the 1200 m shuttle test and the 40 m sprint test will produce greater distances covered at various intensities during EPL match-play. This could impact how practitioners recruit and train players to meet the physical demands of the required game model.

## MATERIALS AND METHODS

### Participants

A squad (n = 21) of full-time professional 1^st^ team male football players (age 29.8 ± 3.4 years; height 183.7 ± 5.2 cm; weight

83.7 ± 6.9 kg) were recruited to participate in this study. The inclusion criteria for the study has been previously employed [28] and included: (i) listed on the roster of the first-team squad of the club at the start of each season, (ii) did not participate in another training program during this study, (iii) completed 75-minutes of playing time when selected. Additionally, the exclusion criteria for the study have also been previously utilised [28] and included: (i) long-term (three months or longer) injury player data, (ii) joining the team late in the study seasons, (iii) an in-sufficient number of satellite connection signals, and (iv) goalkeepers, due to the variations in the physical demands with outfield players [10]. The methodology of differentiating specialised positions was adapted from previous research [29], as various situational factors have an influence on the style of play that can be modulated by different tactical roles [30]. All participants examined were classified based on the regular playing position adopted at the start of each season and remained consistent throughout the study period: Centre back (CB; n = 4), full back (FB; n = 4), centre midfielders (CM; n = 5), wide midfielders (WM; n = 4), and strikers (ST; n = 4).

All data was gathered as a condition of employment in which players are routinely monitored over the course of the competitive season [24], however club approval was obtained. Ethical approval was provided by the University of Central Lancashire (BAHSS 646 dated 17/04/2019) and was conducted in accordance with the most recent version of the Helsinki Declaration (2013). To ensure confidentiality, all data were anonymised prior to analysis. Relevant risk assessments and safety protocols were completed and adhered to in accordance with the football governing body, the FA Premier League and the academic institution.

### Data Capture

League match data from the 2019-20 and 2020-21 seasons were recorded and analysed via an Optical Tracking System (OTS) (Second Spectrum^®^, Los Angeles, USA) to report physical performance data. Data were collected via semi-automated HD cameras with a sampling frequency of 25-Hz and positioned around the stadium using a validated protocol [31]. Data collected for analysis included: total distance covered, measured in meters; high-speed running (HSR), distance covered above 5.5 m · s^−1^ measured in meters; sprint distance, distance covered above 7 m · s^−1^ measured in the meters.

Physical data from the 1200 m shuttle test and 40 m sprint test was monitored using a 18 Hz Global Positioning System (GPS) technology tracking system (Apex Pod, version 4.03, 50 g, 88 × 33 mm; STATSports; Northern Ireland, UK) that has been previously validated for tracking distance covered and MSS during simulated team sports circuits and linear sprinting [32]. All devices were activated 30-minutes before data collection to allow the acquisition of satellite signals and to synchronise the GPS clock with the satellite’s atomic clock [31]. The present GPS system has previously reported excellent inter-unit reliability [33]. Furthermore, the Apex units have shown good levels of accuracy in sport specific metrics in addition to non-significant and trivial differences when measuring MSS against the gold standard measure (Stalker ATS 2.34.7 GHz, United States) [32]. Specifically designed vests were used to hold the devices, located on the player’s upper torso, and anatomically adjusted to each player, as previously described [34]. The GPS signal quality and horizontal dilution of position was connected to a mean number of 21 ± 3 satellites, range 18-23, while HDOP was 0.9. On completion of each session, GPS data were extracted using proprietary software (Apex version 4.3.8, STATSports; Northern Ireland, UK) as software-derived data is a more simple and efficient way for practitioners to obtain data in an applied environment, with no differences reported between processing methods (software-derived to raw processed) [35]. The dwell time (minimum effort duration) was set at 0.5-s to detect high-intensity running and 1-s to detect sprint distance efforts, in-line with manufacturers recommendations and default settings to maintain consistent data processing [34]. Finally, the internal processing of the GPS units utilised the Doppler shift method to calculate both distance and speed data which is shown to display a higher level of precision and less error compared with data calculated via positional differentiation [36].

### Protocol

#### 1200 m Shuttle test

**FIG. 1:**
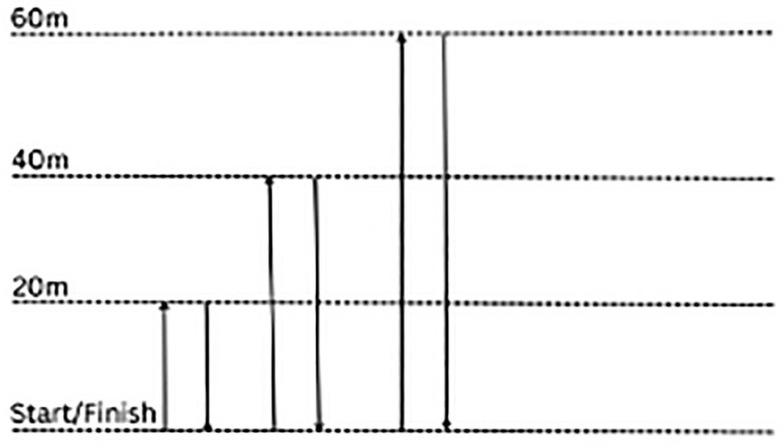
Relationship between total distance covered during match-play and V1.2ST.

During each pre-season period (July), average velocity values were calculated from the 1200 m shuttle test (V1.2ST). The 1200 m shuttle test has previously shown a strong correlation with other field-based MAS tests [17, 37]. Briefly, the test protocol started with poles set at the start point, 20 m, 40 m and 60 m. Players were instructed to run from the start point to the 20 m pole and return to the start point, then to the 40 m pole and return to the start point before running to the 60 m pole and returning to the start point (See Figure 1 for test protocol). This sequence was repeated as quickly as possible five consecutive times until the distance of 1200 m had been completed [17]. To ensure players were performing maximally, players were informed of the remaining test duration at 1-minute intervals until test completion [38]. This verbal encouragement has been shown to be a motivational requirement for laboratory assessments of time to exhaustion and central fatigue [39]. Due to the change of direction within the test, a corrective equation was used: 1200/(Time – 20.3-s (0.7-s for each turn) – V1.2ST (m · s^−1^) [40]. This test was repeated 6 months later in January at the mid-point of each season to allow V1.2ST values to be reviewed.

### 40 m sprint test

During each pre-season period (July) a linear speed phase was completed that consisted of two maximal sprint exposures. This consisted of a 40 m maximal sprint where players were instructed to accelerate maximally from a standing start and sprint as fast as possible to the 40 m marker, at which point players were instructed to decelerate. Following this, each player’s Peak sprinting speed (PSS) reached during this period was established using GPS (Apex, STATSports, Ireland). The researchers decided to take the maximum speed from this period. Average PSS per session may be influenced by session content and positional demands and therefore would not be a true reflection of the players capacity. The 1200 m shuttle test and PSS protocols were previously utilised by Kavanagh et al. [41] to determine a soccer player’s V1.2ST and PSS. The difference between the PSS and V1.2ST measures (ASR1.2ST) were then calculated. The 30%ASR1.2ST measure was employed as previous reported [10, 41]. The 30%ASR1.2ST measure combines V1.2ST and ASR1.2ST to ensure that a reduction in V1.2ST would not result in an increase in the ASR1.2ST measure used [10].

30%ASR1.2ST−(V1.2ST+ASR1.2ST(0.3))

### Statistical analysis

A total of 814 individual match data points were examined with a median of 47 data points per player (range 3 to 74). This resulted in 633 full or nearly full match data points for all players with a median of 39 per player (range 3 to 74). The overall relationship between V1.2ST, 30%ASR1.2ST and distances covered at varying intensities in EPL matches was first assessed by Pearsons correlation coefficient followed by the application of a series of independent Linear Mixed Effects (LME) models, examining the influence of two predictor variables (V1.2ST or 30%ASR1.2ST and position) on three distinct response variables (total distance, HSR and sprint distance). The magnitude of correlations was defined by the following criteria: trivial (r ≤ 0.1), small (0.1 < r ≤ 0.3), moderate (0.3 < r ≤ 0.5), large (from 0.5 < r ≤ 0.7), very large (0.7 < r ≤ 0.9), and almost perfect (r > 0.9) [42]. The first fitted LME models included the player as a random effect. For all combinations of predictor variables and response variables this addition resulted in a significantly better model compared to the simple correlation coefficient, measured in terms of Likelihood Ratio Test (p < 0.001). The model was further improved by adding playing position as a fixed effect. The addition of a fixed effect resulted in a significantly better model, measured in terms of Likelihood Ratio Test (p < 0.001).

Hypothesis testing was conducted to determine if significant differences existed between the constructed model and a model which makes random predictions. Descriptive statistics are reported as mean and standard deviation. All analyses were performed using the R programming language (version 4.3.2). Significance was set at p = 0.05.

## RESULTS

**FIG. 2:**
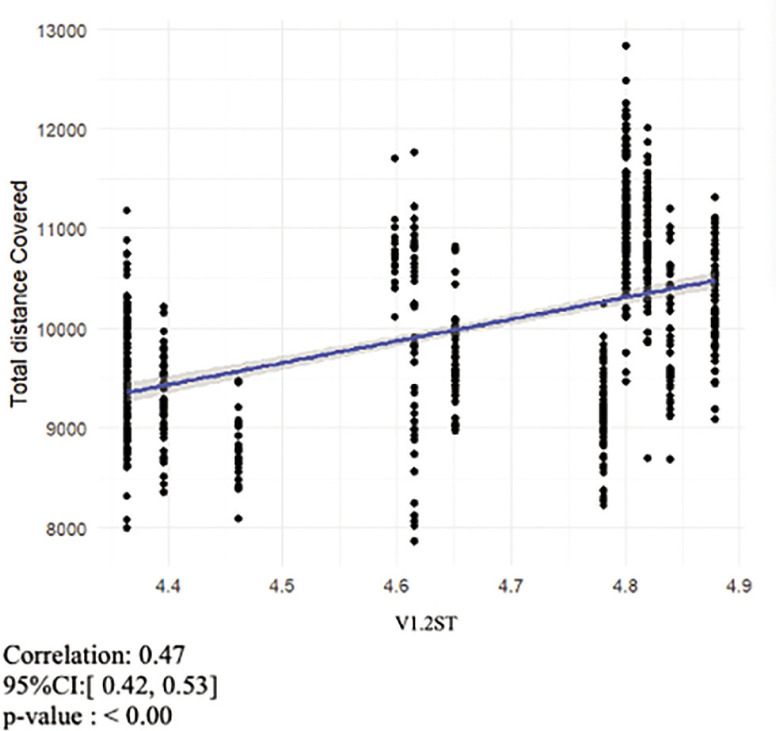
Relationship between total distance covered during match-play and V1.2ST.

**FIG. 3:**
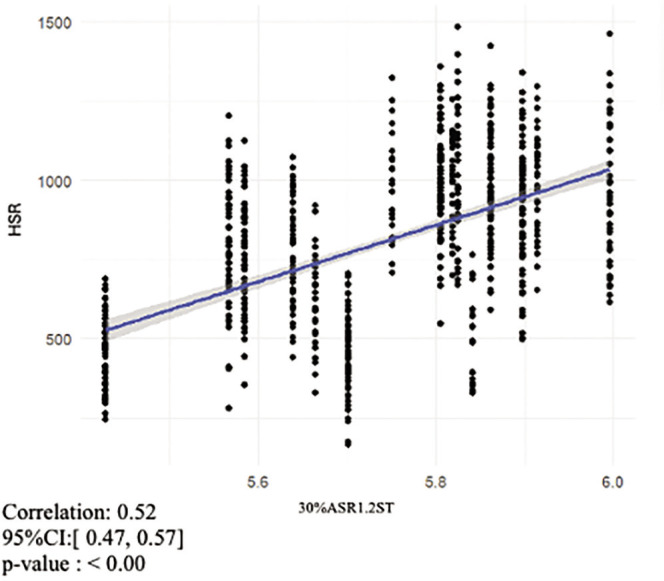
Relationship between HSR distance covered during match-play and 30%ASR1.2ST.

**FIG. 4:**
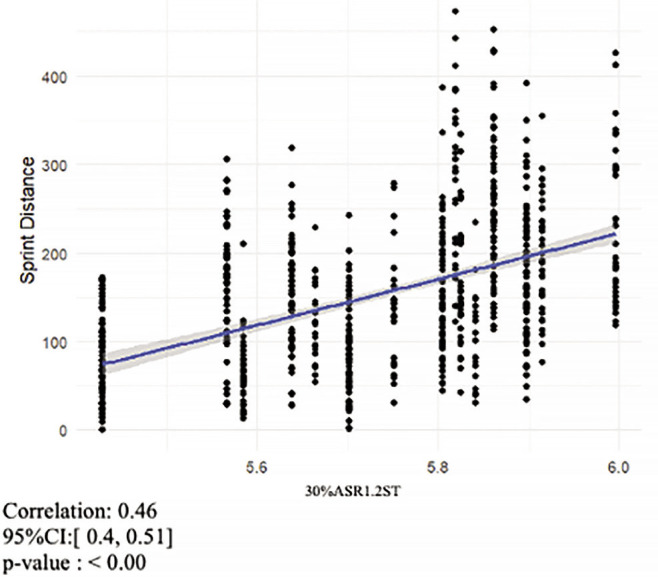
Relationship between sprint distance covered during match-play and 30%ASR1.2ST.

The mean (± SD) V1.2ST value was 4.65 ± 0.20 m · s^−1^, and the mean (± SD) PSS value was 9.53 ± 0.38 m · s^−1^. Mean (± SD) values for ASR1.2ST and 30%ASR1.2ST were 4.88 ± 0.44 m · s^−1^ and 5.77 ± 0.15 m · s^−1^ respectively. The mean (± SD) total distance covered was 9985.05 ± 910.79 m, the mean (± SD) HSR distance covered was 805.05 ± 261.97 m and the mean (± SD) sprint distance covered was 155.16 ± 87.12 m. The correlation coefficients (Figure 2-4) for the overall relationship between V1.2ST and total distance covered and 30%ASR1.2ST and sprint distance covered were moderate (r = 0.47 and 0.46 respectively). The correlation coefficient for the overall relationship between 30%ASR1.2ST and HSR distance covered was large (r = 0.52). All other relationships displayed trivial to small correlations. The overall relationship between 30%ASR1.2ST and total distance covered was r = 0.32, while the relationship between V1.2ST and HSR was r = 0.25. The correlation coefficients for the relationship between V1.2ST and SD was r = 0.02.

Displayed in Table 1 for every increase of one unit in 30%ASR1.2ST there was a 495 m ± 173 m increase in the HSR distance covered, which is statistically significant (p = 0.02). Every increase of one unit in 30%ASR1.2ST was associated with a 209 m ± 80 m increase in the SD covered, which is statistically significant (p = 0.02). All other relationships were not significant although, all dispalyed a positive linear relationship.

**TABLE 1:** Statistical results when fitting LME model

Independent variable	Dependant variable	Estimate	Std. Error	t value	p value
V1.2ST	Total Distance Covered	1032.66	516.01	2.00	0.07
30%ASR1.2ST	Total Distance Covered	883.86	651.14	1.35	0.20
V1.2ST	High-speed running distance	250.34	185.20	1.35	0.20
30%ASR1.2ST	High-speed running distance	495.47	173.03	2.86	0.02*
V1.2ST	Sprint distance	55.46	88.15	0.63	0.54
30%ASR1.2ST	Sprint distance	209.06	80.24	2.60	0.02*

## DISCUSSION

The aim of this research was to investigate the relationship between the physical characteristics of V1.2ST and 30%ASR1.2ST derived from the 1200 m shuttle test and 40 m sprint test with distances covered at various intensities during EPL matches. The present results indicate that high levels of physical fitness obtained during these tests can improve match running performance in elite soccer matches. Although not statistically significant the results show a positive association with V1.2ST and distance covered (Table 1). Indeed for every unit increase in V1.2ST, there was an increase of 1032 m in total distance covered, (p = 0.07). Swaby et al. [15] found a strong relationship between MAS and distance covered during elite rugby union matches. The results of this study agree with existing literature that states that high levels of aerobic fitness correlate with greater total distance covered during match-play [43, 44]. This study is the first to find similar results using the 1200 m shuttle test in an EPL population.

The 30%ASR1.2ST does not seem to be a significant predictor of the total distance covered (Table 1), indicating that the relationship between 30%ASR1.2ST and distance covered may not be as strong as the one between V1.2ST and distance covered. Most notably, 30%ASR1.2ST has a significant effect on HSR (p = 0.02) and sprint distance (p = 0.02) covered during match-play. This may be due to the demands of soccer that require elite level players to have well developed anaerobic energy systems [45]. This information is invaluable to sports science practitioners and coaches who may look to increase the 30%ASR1.2ST of players in order to increase HSR and sprint output during competitive matches. This could be achieved by improving either V1.2ST or PSS. Ortiz et al. [24] found players with a higher ASR presented greater sprint demands and reached greater MSS when compared to players with lower ASR. Collison et al. [22] found that that prescription by ASR reduces the variability in supramaximal interval running performance in comparison to prescription by MAS. This reduction in variability ensures all athletes are exposed to similar physiological demands, and in turn similar physiological adaptations [22]. This provides practitioners with information which may influence coaching and recruitment. For example, a high-intensity transition-based game model may require players that can tolerate more HSR distance (> 5.5 m · s^−1^) than the opposition. Practitioners could use the 1200 m shuttle test and PSS testing to identify players who fit the team’s game model. Indeed, specific positions may require different locomotor profiling.

Although not statistically significant a unit increase in V1.2ST was associated with an increase of 250 m in HSR distance. The key aspect to consider is the practical significance of the reported small differences in physical outputs [36]. Current literature has paid particular attention to these high-speed physical metrics to guide training approaches to optimise performance [14]. While knowledge of this information allows practitioners to tailor training load based on each players individual haracteristics, thus potentially increasing performance.

## CONCLUSIONS

Despite the strengths of this study, there are some limitations to list, including a) the study was conducted using only one team and thus a limited sample of players were examined, which consequently may restrict a generalisation of the results; b) V1.2ST was only tested twice during each season; c) contextual factors such match location, score status, and team formation [46, 47] were not considered in this study, this may potentially influence the positional demands over the course of a match [4]. Future research should aim to re-test V1.2ST at various stages of the season to ensure that individualised speed thresholds represent the players physical characteristics throughout the season. In addition, practitioners should examine the relationship between different locomotor profiles, playing position and distances covered at high intensities. This may provide practitioners with valuable information with regards to team selection, player recruitment and energy system development.

## Practical Applications

This research allows practitioners to identify physical match outputs using the 1200 m shuttle test. Indeed, players with a higher 30%ASR1.2ST showed greater absolute HSR and sprint performance. This may impact coaching and recruitment strategies. For example, a high-intensity transition-based game model may require players that can cover more high-intensity distance than the opposition. Therefore, 1200 m shuttle and 40 m sprint tests may be beneficial to identify the players that fit the specific teams game model. This further illustrates the importance of individualised HSR thresholds to aid the training approach relative to each individual athletes profile. A better understanding of these player profiles and the physical limitations that exist may offer important information for coaches aiming to execute a particular playing style. These results may assist performance practitioners in selecting the most adequate post-match recovery strategies or training models based on the player’s individualised characteristics.
